# Medical Convention

**Published:** 1847-06

**Authors:** 


### THE WESTERN JOURNAL.

New Series.—Vol. VII.—No. VI.

LOUISVILLE, JUNE 1, 1847.

### MEDICAL CONVENTION.

We lay before our readers such details as have reached us of the proceedings of the Medical Convention in Philadelphia. They will be read with deep interest. In our next number we shall be able to present a full account of the meeting.

The following resolutions were adopted:

"1. That it be recommended to all the colleges to extend the period employed in lecturing, from four to six months.

"2. That no student shall be a candidate for the degree of M.D. unless he shall have devoted three entire years to the study of medicine, including the time allotted to attendance upon the lectures.

"3. That the candidate shall have attended two full courses of lectures; that he shall be twenty-one years of age; and in all cases shall produce the certificate of his preceptor to prove when he commenced his studies.

"4. That the certificate of no preceptor shall be received who is avowedly and notoriously an irregular practitioner, whether he possess the degree of M.D. or not.

"5. That the several branches of medical education already named in the body of this report be taught in all the colleges, and that the number of professors be increased to seven.

"6. That it be required of candidates that they shall have steadily devoted three months to dissections.

"7. That it is incumbent upon preceptors to avail themselves of every opportunity to impart clinical instruction to their pupils; and that medical colleges require candidates for graduation to show that they have attended on hospital practice for one season, whenever it can be accomplished, for the advancement of the same end.

"8. That it is incumbent upon all schools and colleges granting diplomas, fully to carry out the above requisitions.

"9. That it be considered the duty of preceptors to advise their students to attend only such institutions as shall rigidly adhere to the recommendations herein contained.

"10. That it be suggested to the faculties of the several medical institutions to adopt some efficient means for ascertaining that their students are actually in attendance upon their lectures."

The registration of the births, deaths and marriages, by the several States, was one of the subjects introduced a year ago which occupied the attention of the Convention. The committee appointed at the previous meeting made a report concerning it, and submitted the following resolutions, which were adopted:

"1. That it is expedient for this Convention to recommend to and urge upon the various State Governments the adoption of measures for procuring a registration of the births, marriages and deaths occurring in their several populations.

"2. That a standing committee be appointed by the Convention to take a general charge of the subject, and report annually to the Convention.

"3. That the State medical societies be requested to assume the duty of carrying out the objects embraced in the first resolution; and that in those States where no organized societies exist, the delegates therefrom in the present Convention be charged with the duty for their respective States, and report to the standing committee.

"4. That in procuring the registration, the forms and nomenclature adopted should be as nearly as possible similar to those prepared for and reported to the Convention.

"5. That the paper hereto annexed be adopted as the voice of the Convention, be printed, and signed by its officers, and transmitted under their direction to all the State Governments of the Union."

The Convention before adjourning resolved itself into an association, to be called the 'American Medical Association,' and elected the officers of the same. The following gentlemen were chosen: Professor Chapman, President; Prof. Knight, of New Haven, Dr. A. Stevens, of New York, Dr. Buchanan, of Nashville, and Dr. Moultrie, of South Carolina, Vicepresidents; Dr. Stillé, of Philadelphia, and Dr. Dunbar, of Baltimore, Secretaries; and Dr. Hays, of Philadelphia,

Treasurer. The president delivered a brief address, of which we find the following report in a Philadelphia paper:

"The committee, accompanied by Dr. Chapman, the president, came into the association, when the members immediately rose and applauded him as he proceeded to the rostrum. Having ascended it, the doctor briefly addressed the association, saying he was greatly impressed with the honor conferred upon him, and unable to express in adequate terms the feelings which then animated his breast. It had been his good fortune, in the course of his long life, sometimes to have been complimented in the same manner, though not in the same degree. He confessed his incompetency to serve them in conformity with his wishes. He loved his profession, and should be very ungrateful did he not. Whatever he possessed in this life of any value had been bestowed by her great favors. Whenever he deserted her or any of her disciples, might Almighty God desert him! It would always be his aim to promote as far as he could the principles of the science, and do everything in his power to elevate still higher the medical profession.

"In a few minutes afterwards the Convention adjourned, to meet again in May next, at Baltimore."

